# Investigating the Influence of Oxford Unicompartmental Knee Arthroplasty Keel on Sagittal Plane Stresses: A Finite Element Analysis

**DOI:** 10.7759/cureus.86506

**Published:** 2025-06-21

**Authors:** Takaaki Imada, Mitsuru Hanada, Kohei Murase, Yukihiro Matsuyama

**Affiliations:** 1 Department of Orthopedic Surgery, Hamamatsu University School of Medicine, Hamamatsu, JPN; 2 Center for Industry-University Collaboration, Graduate School of Engineering Science, Osaka University, Osaka, JPN

**Keywords:** finite element analysis, keel, knee osteoarthritis, sagittal plane, unicompartmental knee arthroplasty

## Abstract

Purpose

The specific impact of keel placement and tibial implant position on stress reduction and fracture risk in the sagittal plane of the Oxford unicompartmental knee arthroplasty (UKA) system remains unclear. Therefore, this study aimed to investigate the stresses produced by the Oxford UKA keel in the sagittal plane to better understand their effects on fracture risk and optimize surgical outcomes.

Methods

This retrospective study included 89 patients who underwent Oxford UKA. The effects of the position and size of the tibial component on the anterior and posterior cortical distances were assessed using postoperative sagittal radiographs. Finite element analysis was performed using three-dimensional surface models of the trabecular and cortical bones of the tibia created from preoperative computed tomography-Digital Imaging and Communications in Medicine data.

Results

Clinical analysis data showed a correlation between the posterior placement of the tibial component and the distance between the posterior cortex and keel. The smaller size of the tibial component predominantly reduced the distance between the posterior cortex and the keel. Finite element analysis showed that moving the medial loading point of the tibia posteriorly increased the keel’s exposure to posterior cortical stresses. Posterior displacement of the tibial component and additional posterior transection of the keel further increased the stresses in the tibial diaphyseal cortex.

Conclusion

Posterior placement and small size of the tibial component, as well as posterior osteotomy of the keel, were associated with increased stresses in the sagittal plane resulting from the Oxford UKA keel.

## Introduction

Knee arthroplasty is a widely used surgical procedure for patients with knee osteoarthritis (KOA), with the aim of improving pain relief and restoring functionality. Among the various options available, Oxford unicompartmental knee arthroplasty (UKA) has gained popularity because of its potential advantages, such as preserving healthy tissue, maintaining ligament stability, and enabling more natural knee kinematics [1,2]. Understanding the biomechanical factors that influence the success and longevity of knee implants is crucial for optimizing patient outcomes and implant designs.

A key aspect affecting the stability of the Oxford UKA system is the stress distribution within the implant and the surrounding bone tissues. The keel component plays an important role in distributing the load and firmly holding the implant in place. It is essential to consider the interaction between the keel and bone tissues, as changes in stress distribution can affect bone remodeling and implant stability [3]. Previous studies have identified potential concerns related to the posterior cutting of cancellous and cortical bones during keel resection, which may increase the fracture risk at the bone-implant interface [4].

Furthermore, placement of the tibial component in knee arthroplasty is crucial to obtain satisfactory outcomes. Posterior placement of the tibial component may interfere with the natural force transmission, compromise the integrity of the bone structure, and increase the fracture risk. Although the influence of keel placement and tibial implant positioning on fracture risk has been examined in various contexts, to the best of our knowledge, no previous study has comprehensively assessed the influence of these specific factors on stress distribution and fracture risk in the sagittal plane of the Oxford UKA system [5,6].

Therefore, in this study, we aimed to illustrate the stress effects caused by the Oxford UKA keel in the sagittal plane using a finite element analysis approach. By analyzing different scenarios, including posterior cutting of cancellous and cortical bones along the keel and posterior placement of the tibial components, we aimed to investigate the potential associations between increased fracture risk and implant stability.

## Materials and methods

The study included 89 patients who underwent primary UKA for KOA and spontaneous osteonecrosis of the knee between April 2020 and July 2023. The patients and their families provided consent after being informed that data from the research would be submitted for publication. Oxford UKA was performed in all patients. The study was conducted in accordance with the relevant national regulations, institutional policies, and the tenets of the Helsinki Declaration and has been approved by the JA Shizuoka Koseiren Enshu Hospital Ethics Review Committee (approval number: 2025-05-02).

**Figure 1:**
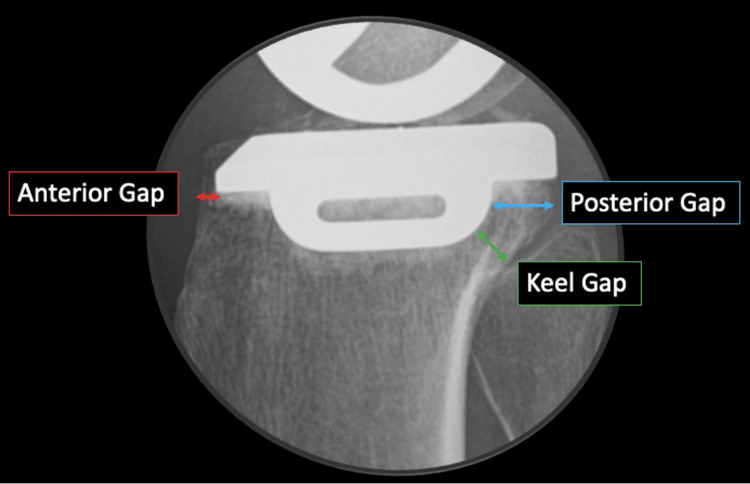
Postoperative radiographs (lateral view) of the patients Anterior gap: distance from the anterior tibial cortical bone to the tibial component. Keel gap: the shortest distance from the posterior margin of the keel to the posterior cortical bone. Posterior gap: horizontal distance from the posterior margin of the keel to the posterior cortical bone.

The measurements included a pre- and postoperative range of motion (ROM), Japanese Orthopaedic Association (JOA) score, Knee Society Score (KSS), and the hip-knee-angle (HKA). The distance from the anterior tibial cortex to the component (anterior gap), the shortest distance from the posterior margin of the keel to the posterior cortical bone (keel gap), and the horizontal distance from the posterior margin of the keel to the posterior cortical bone (posterior gap) were measured from sagittal radiographs of each patient, and their relationships were investigated (Figure 1).

Bone cut, model creation, and prosthesis assembling

A three-dimensional (3D) nonlinear finite element knee joint model was created based on computed tomography (CT) images (per 1 mm slice) of an 84-year-old woman with KOA.

**Figure 2:**
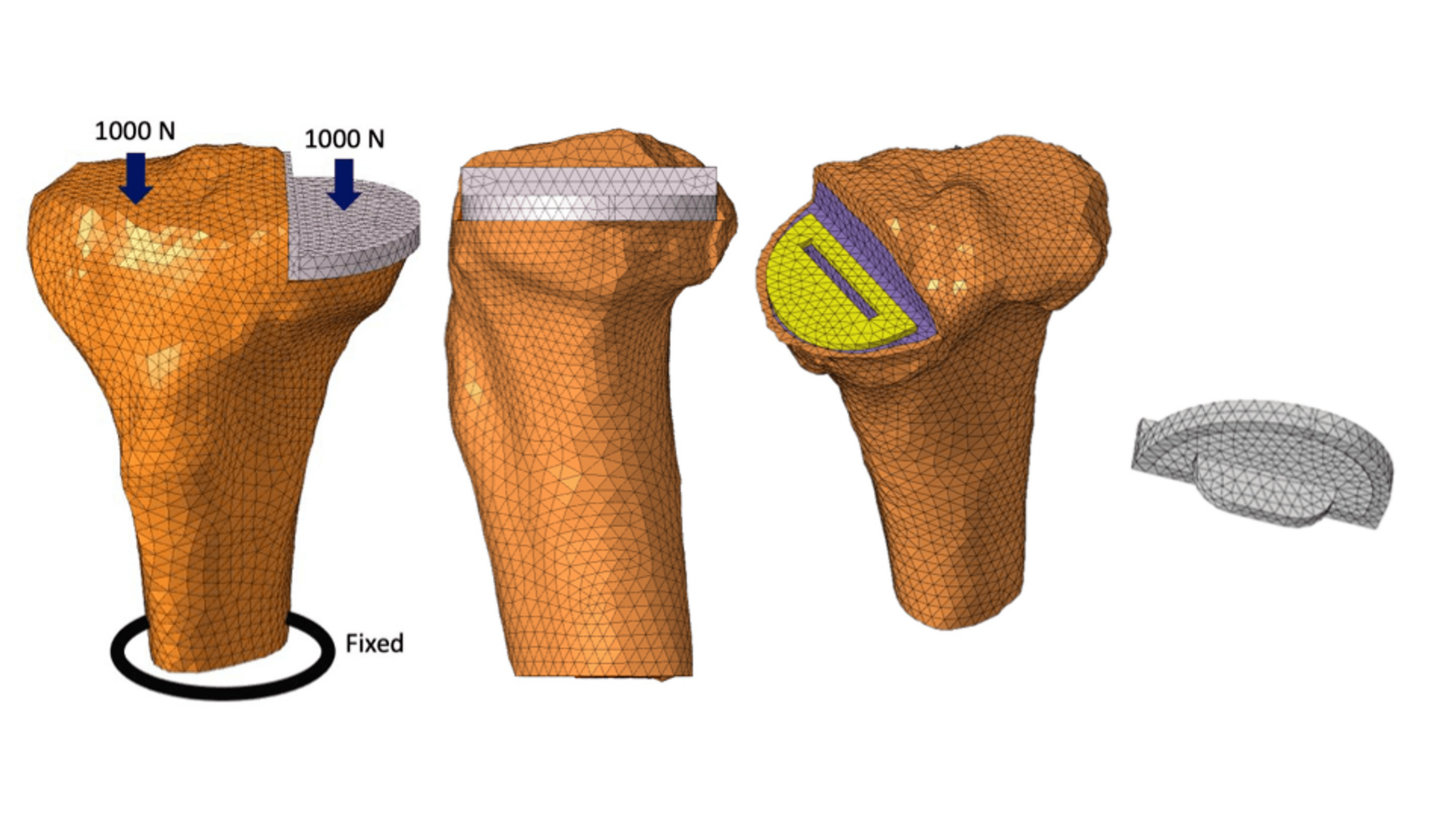
Unicompartmental knee arthroplasty (UKA) load conditions A force of 1000 N is added to each of the medial and lateral tibial articular surfaces parallel to the tibial axis. The tibial diaphysis is fixed. Image Credits: Dr. Takaaki Imada

**Figure 3:**
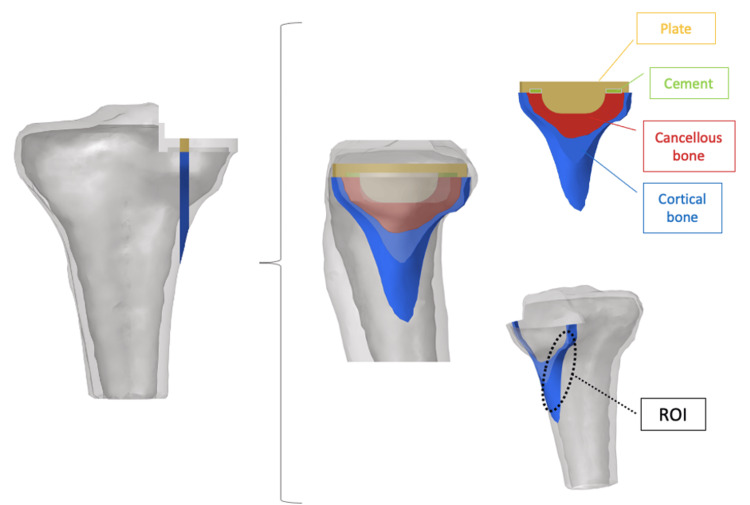
Evaluating equivalent stress in the proximal tibia The tibia is cut in the sagittal plane to match the width of the keel. The ROI of the proximal tibia in the quantitative equivalent stress (von Mises stress) measurements is defined as the posterior region 15 mm below the articular surface of the medial tibial condyle of the knee joint. ROI: region of interest Image Credits: Dr. Takaaki Imada

This computational knee joint model has been established and validated in previous studies [7,8]. A 3D surface model of the trabecular and cortical bone of the tibia was created from the preoperative CT-Digital Imaging and Communications in Medicine data of the patient using 3D model software (Mimics Ver. 14; Materialise, Leuven, Belgium) [9-11]. The model was then divided into elements using finite element modeling software (Computer-Aided Engineering (CAE); HyperWorks, Troy, MI, USA) to create a finite element model consisting of 60,086 secondary elements of a tetrahedron. The tibial component was positioned based on the preoperative CT data using ZedKnee (Lexi, Tokyo, Japan). CAE was used to model the same size as the positioned Oxford UKA size A (anteroposterior diameter: 45.2 mm; mediolateral width: 26.0 mm). The tibial component position was defined as 0° varus in the coronal plane, 5° posterior tilt in the sagittal plane, and neutral rotation according to Akagi's line. The tibial component was positioned according to the criteria described in the manufacturer’s surgical technique guide (Oxford Partial Knee Microplasty Instrumentation Surgical Technique). The cement thickness was set as 1 mm. The tibial components were fully bonded to the tibia to simulate cement use. The fixation plane was the tibial diaphysis, 90 mm distal to the apex of the medial intercondylar spine [12]. Assuming the weight of the patient (approximately 40 kg) and the load on the knee when standing on one leg (approximately five times the body weight), 1000 N was applied to each of the medial and lateral tibial articular surfaces parallel to the tibial axis [13-15]. The medial loading point was defined as the tibial component surface relative to the center of the femoral component, and the lateral load point was defined as the midpoint of the lateral femoral condyle (Figure 2) [16]. Each material is assumed to be a linear elastic body. Table 1 lists the material constants (Young's modulus and Poisson's ratio) [12,17]. The region of interest of the proximal tibia in the quantitative equivalent stress (von Mises stress (VMS)) measurements was the posterior region, 15 mm below the articular surface of the medial tibial condyle of the knee joint (Figure 3) [13].

**Table 1:** Material constants CoCrMo: Cobalt-chromium-molybdenum

Material	Young’s modulus (MPa)	Poisson’s ratio
Cortical bone	5,000	0.3
Cancellous bone	1,000	0.25
Cement	500	0.3
CoCrMo alloy	200,000	0.3

Tibial component placement and keel cut

**Figure 4:**
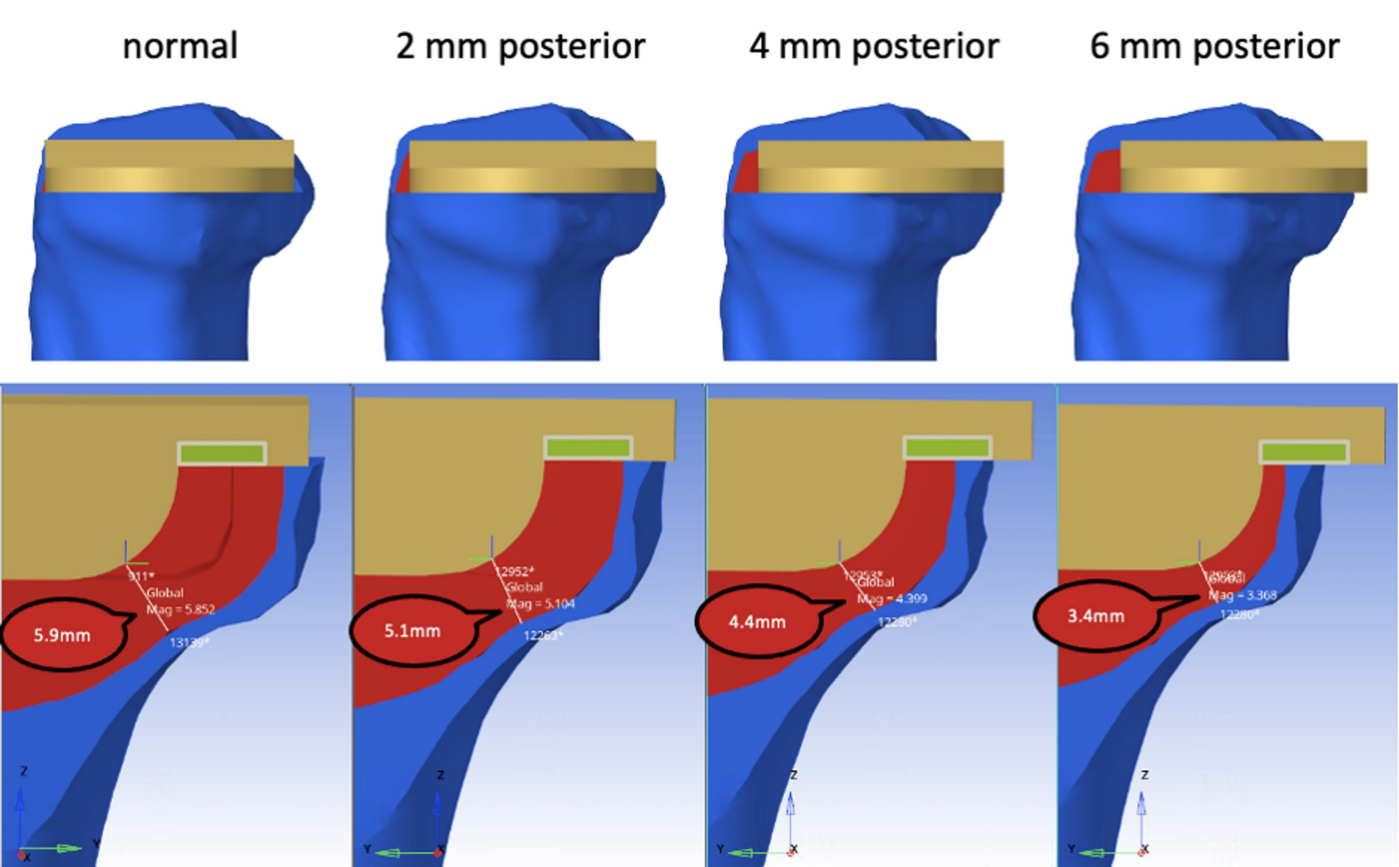
Tibial component placement Tibial component placement according to the preoperative plan, from which the component placement is moved 2 mm posteriorly (keel gap: normal, 5.9 mm; 2 mm posterior, 5.1 mm; 4 mm posterior, 4.4 mm; 6 mm posterior, 3.4 mm).

**Figure 5:**
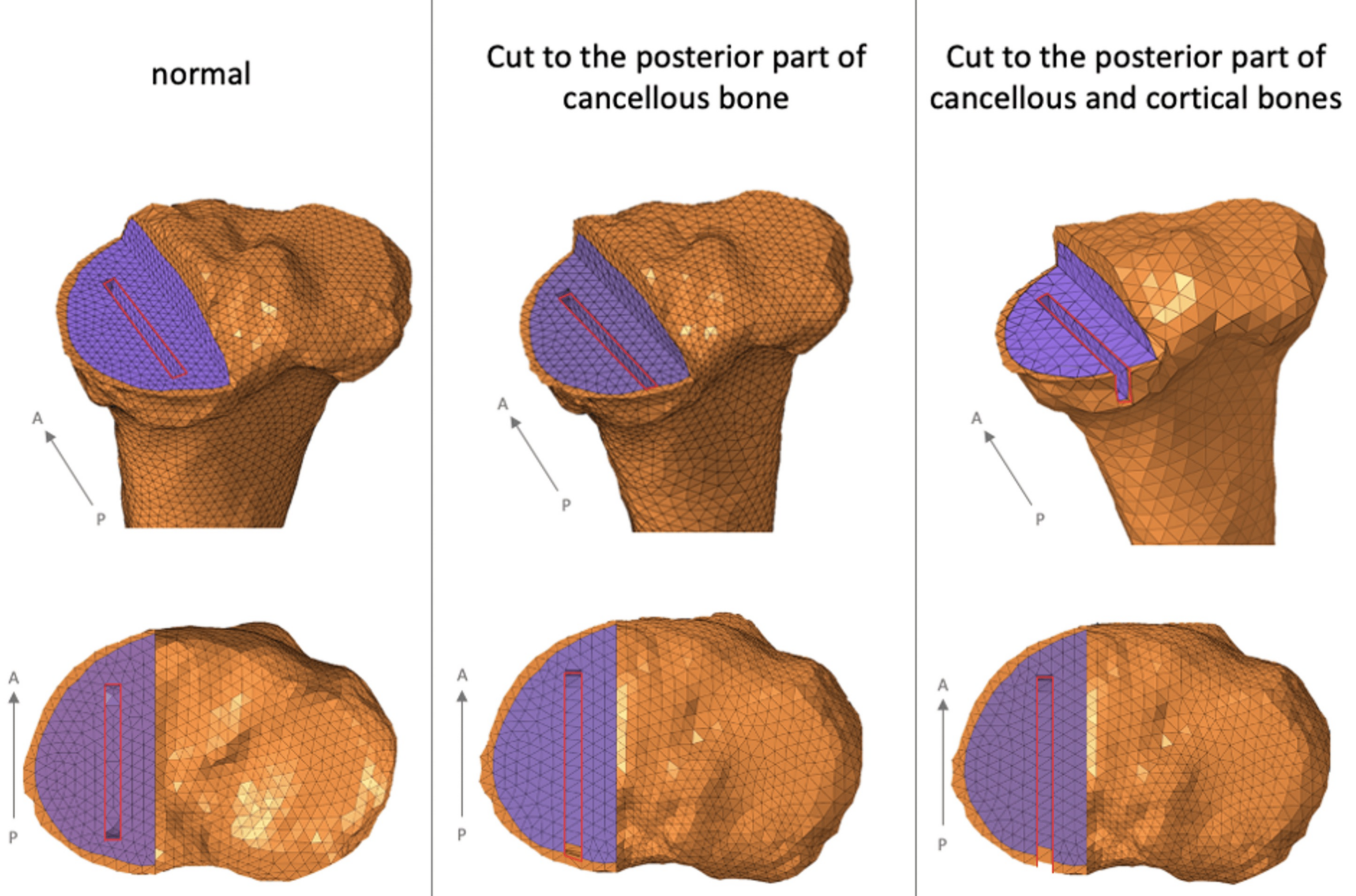
Keel cut further posteriorly When cutting the keel along the osteotomy guide, or to the posterior part of the cancellous bone, or to the posterior part of the cancellous and cortical bone.

In the present study, we investigated various scenarios regarding tibial component placement. First, we examined cases where the tibial component was positioned according to the preoperative plan and then moved posteriorly by 2 mm. The keel gap measurements for these positions were as follows: normal placement, 5.9 mm; 2 mm posterior, 5.1 mm; 4 mm posterior, 4.4 mm; 6 mm posterior, 3.4 mm (Figure 4). Additionally, we assessed the impact of further posterior transection of the keel, with cuts made to the posterior parts of the cancellous bone and to both the posterior cancellous and cortical bones (Figure 5). The VMS occurring in the posterior tibial cortical bone was measured and compared under these conditions.

**Figure 6:**
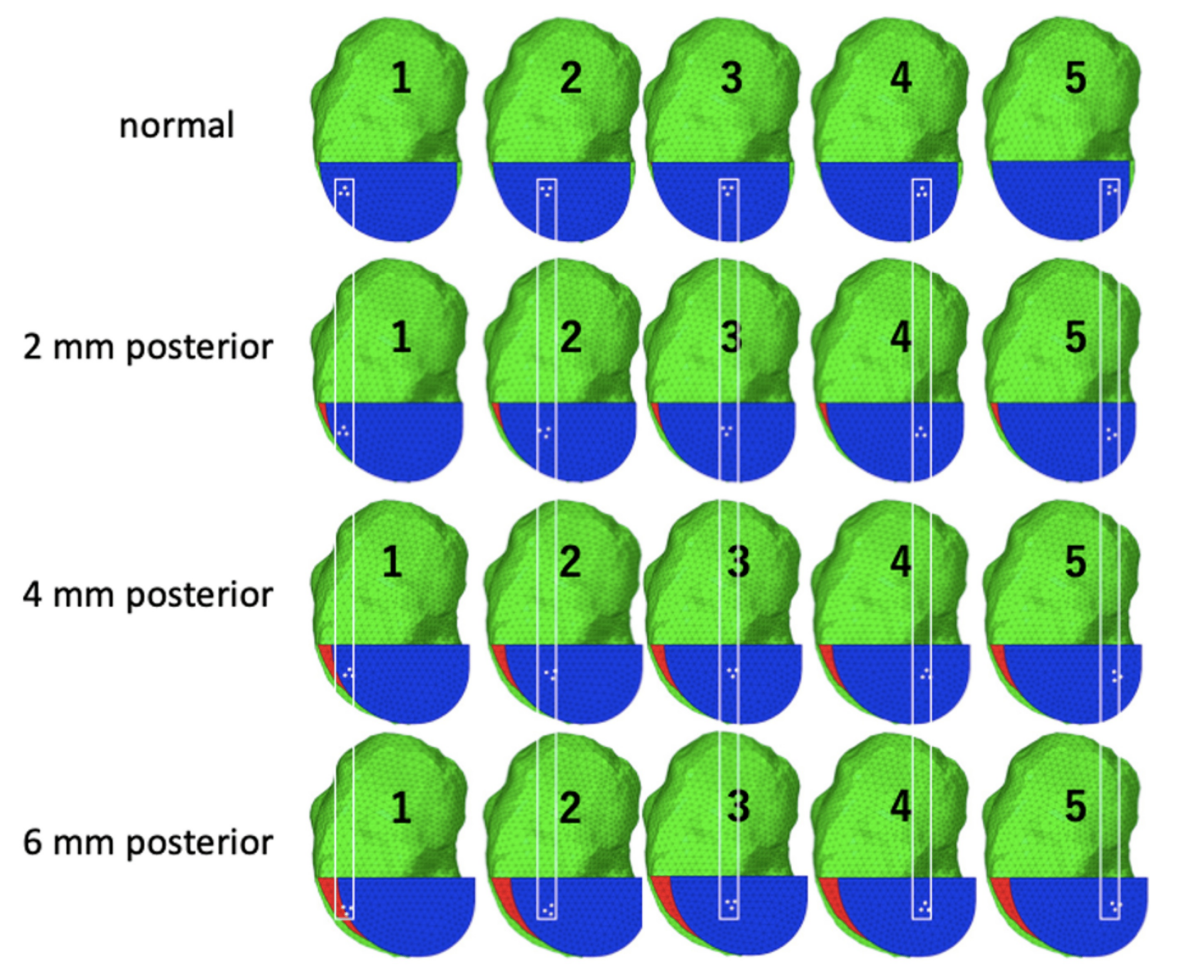
Load position of Oxford UKA Five loading points are set because the Oxford UKA insert is mobile and the loading position moves from anterior to posterior. The loading position is set to be approximately the same in the coronal slice even if the tibial component moved posteriorly. UKA: unicompartmental knee arthroplasty

As the Oxford UKA utilizes a mobile insert, which causes the load position to shift back and forth, we plotted the medial load point from anterior to posterior. Stresses were measured at five locations set in almost identical positions in the coronal slice (Figure 6).

Statistical analysis

All image measurements were conducted by a single experienced orthopedic surgeon (TI). Demographic characteristics of the patients are summarized as means with standard deviations. Student's t-test was used to compare normally distributed data between two groups, whereas the Mann-Whitney U test was applied for analysis of data not normally distributed. The chi-square test was used to compare the outlier rates between the groups. All significance tests were two-tailed, and a significance level was set at p less than 0.05 for all tests. The association between anterior, posterior, and keel gaps on sagittal radiographs was analyzed using Pearson's correlation coefficient. Statistical analysis was performed using IBM SPSS Statistics for Windows, Version 26 (Released 2019; IBM Corp., Armonk, NY, USA).

## Results

Patient characteristics

The background characteristics of the patients are shown in Table 2. Of the 89 patients, 29 were male and 60 were female, with a mean age of 73.9 ± 6 years at surgery. The observation period was 24 months.

**Table 2:** Demographic characteristics of the patients Data are presented in numbers or mean ± standard deviation. HKA: hip-knee-ankle; JOA: Japanese Orthopaedic Association; KL: Kellgren-Lawrence; OA: osteoarthritis; ON: osteonecrosis

Characteristic	Value
Number of cases	89
Sex	
Female	60
Male	29
Diagnosis	
OA	79
ON	10
KL grade	
1	1
2	17
3	37
4	34
Age (years)	73.9 ± 6.4
Height (cm)	152.9 ± 7.8
Body weight (kg)	60.1 ± 9.2
Operation time (min)	73.5 ± 20.1
Hospital time (days)	22.3 ± 9.9
Implant size	
AA	10
A	19
B	30
C	21
D	5
E	4
Knee extension	
Preoperative	5.5 ± 8.9
Postoperative	3.2 ± 3.0
Knee flexion	
Preoperative	122.4 ± 7.2
Postoperative	123.2 ± 7.1
Knee Society Score	
Preoperative	84.2 ± 25.4
Postoperative	116.4 ± 24.9
JOA score	
Preoperative	59.8 ± 11.9
Postoperative	82.3 ± 9.7
HKA (Varus°)	
Preoperative	8.7 ± 5.8
Postoperative	4.1 ± 4.1

Clinical outcomes

**Figure 7:**
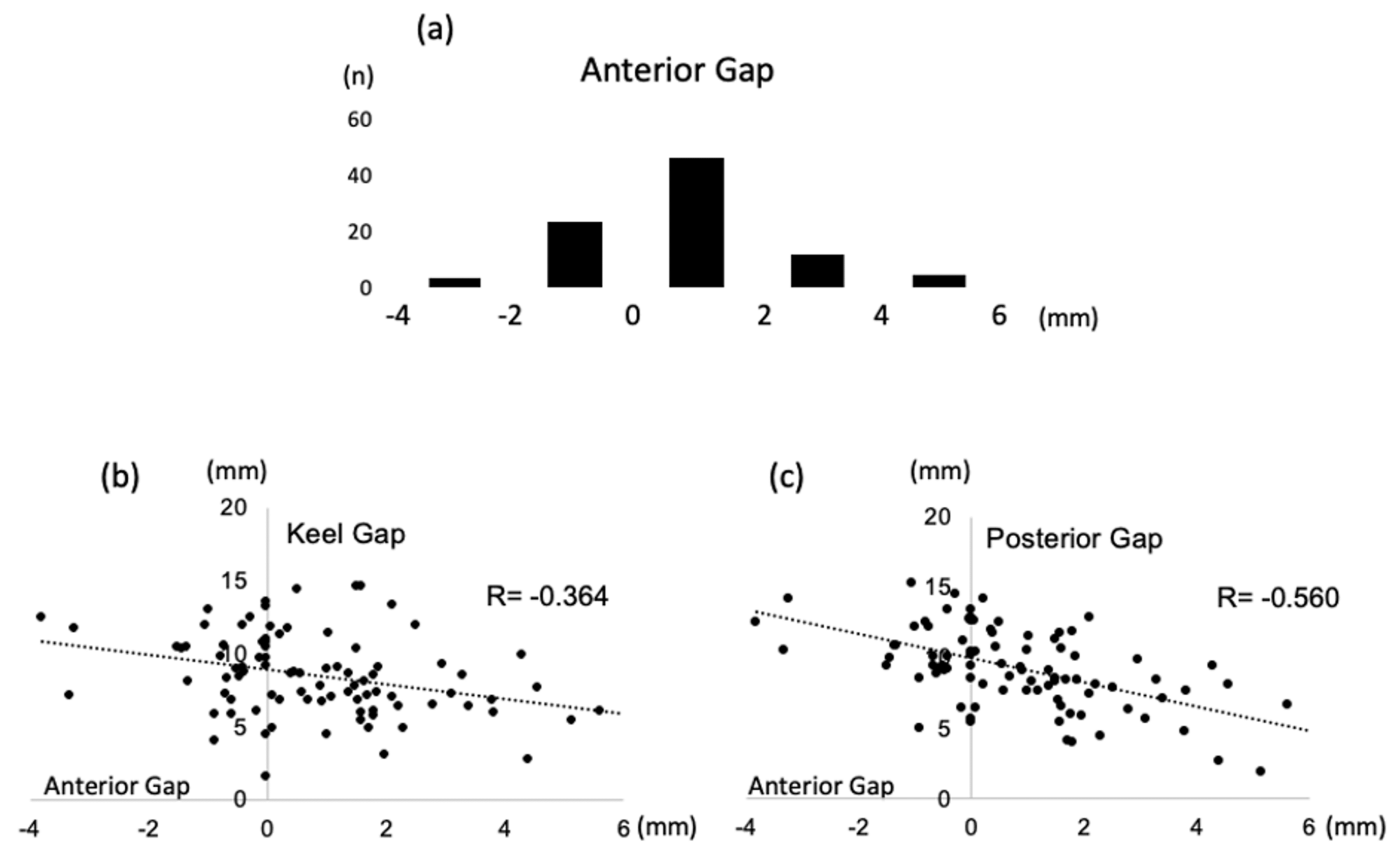
Correlation between tibial component posteriorization and anterior/keel gaps in unicompartmental knee arthroplasty (a) The tibial component tends to be placed posteriorly, with an average placement of 0.8 mm posteriorly. In 19% of all cases, these are placed more than 2 mm posteriorly. (b) Relationship between the anterior and keel gaps; (c) Relationship between the anterior and posterior gaps. Both show negative correlations, indicating that the more the tibial component shifts posteriorly, the shorter the distance between the posterior cortical bone and keel.

The tibial component was placed, on average, 0.8 mm posterior. In 19% of cases, the placement exceeded 2 mm posteriorly (Figure 7a), indicating a tendency for posterior positioning of the tibial component. A negative correlation was observed between the anterior and posterior gaps (r = -0.560, p = 0.000), as well as between the anterior and keel gaps (r = -0.364, p = 0.011). This indicates that as the tibial component is shifted posteriorly, the distance between the posterior cortical bone and keel decreases (Figures 7B-7C). Additionally, the tibial components tended to be relatively small. When comparing by component size, the distance between the keel and posterior cortical bone decreased as the component size became smaller (Table 3). There was a mild negative correlation between the posterior gap and JOA score, but the keel, posterior, and anterior gaps were not correlated with postoperative ROM, JOA score, and KSS (Table 4).

**Table 3:** Oxford UKA tibial component size and sagittal plane gap UKA: unicompartmental knee arthroplasty

Size	AA	A	B	C	D	E
n	10	19	30	21	5	4
Keel gap (mm)	7.1 ± 3.4	7.7 ± 2.1	7.7 ± 2.3	10.3 ± 2.4	10.3 ± 3.2	10.8 ± 3.0
Posterior gap (mm)	8.5 ± 3.8	9.2 ± 2.2	8.3 ± 2.7	10.4 ± 2.5	10.5 ± 2.2	10.6 ± 1.5

**Table 4:** Correlation between the keel position and patient reported outcome measures KSS: Knee Society Score; JOA: Japanese Orthopaedic Association

	Keel gap	Posterior gap	Anterior gap
Postoperative knee extension	-0.049	-0.05	0.083
Postoperative knee flexion	0.193	-0.031	0.073
Postoperative KSS	0.038	-0.085	0.209
Postoperative JOA score	0.035	-0.314	0.246

Load position and stress distribution

**Figure 8:**
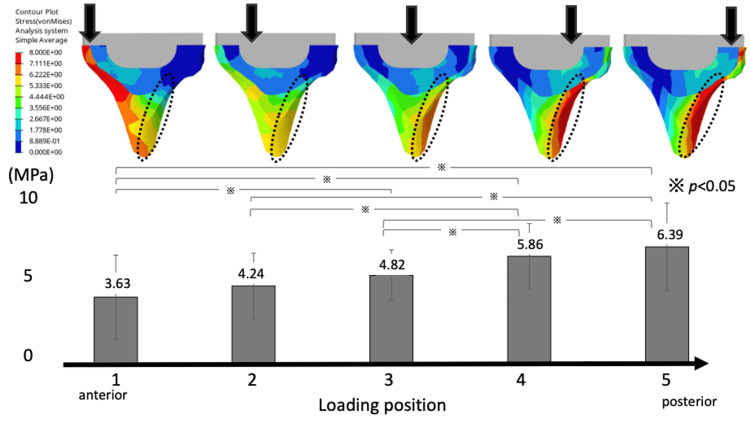
Von Mises stresses in the cancellous and cortical bones in the sagittal section The von Mises stress values are concentrated in the posterior metaphyseal cortex of the tibia below the keel. As the medial loading point of the tibia moves posteriorly, there is a significant increase in the von Mises stress arising from the tibial component to the posterior cortical bone.

The VMS generated in the cancellous bone of the tibia was concentrated in the region immediately below the keel, extending to the cortex of the posterior tibial diaphysis. In addition, as the medial loading point of the tibia moved posteriorly, there was a significant increase in the VMS values arising from the tibial component to the posterior cortical bone (loading position (anterior to posterior): 3.63 ± 2.28, 4.24 ± 1.77, 4.82 ± 1.44, 5.86 ± 1.80, and 6.39 ± 2.35 at positions 1-5, respectively) (Figure 8).

**Figure 9:**
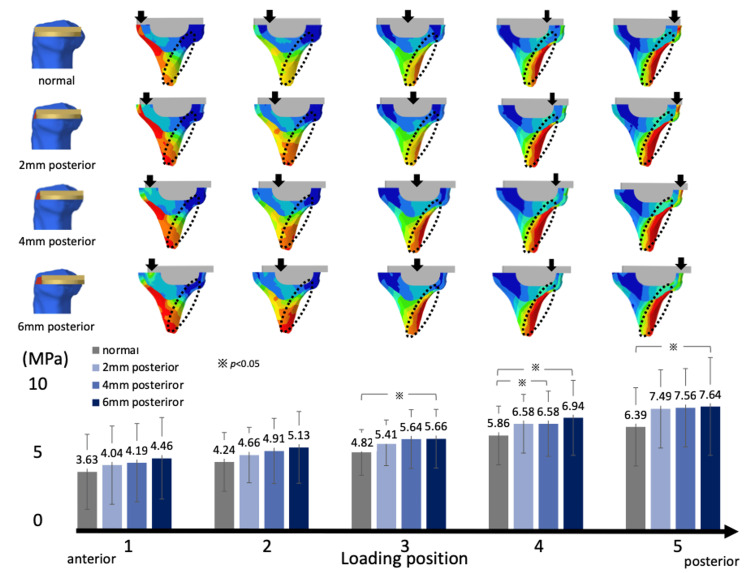
The tibial component is placed posteriorly by 2 mm The von Mises stress values of the posterior cortical bone of the tibia are increased. As the loading position moves posteriorly, the von Mises stress increases.

Additionally, when the tibial component was placed posteriorly by 2 mm, the posterior stresses in the cortical bone of the tibial diaphysis increased (normal: 4.82 ± 1.47, 2 mm posterior: 5.41 ± 1.71, 4 mm posterior: 5.64 ± 1.75, and 6 mm posterior: 5.66 ± 1.80). The stresses tended to increase as the loading position moved posteriorly (Figure 9).

**Figure 10:**
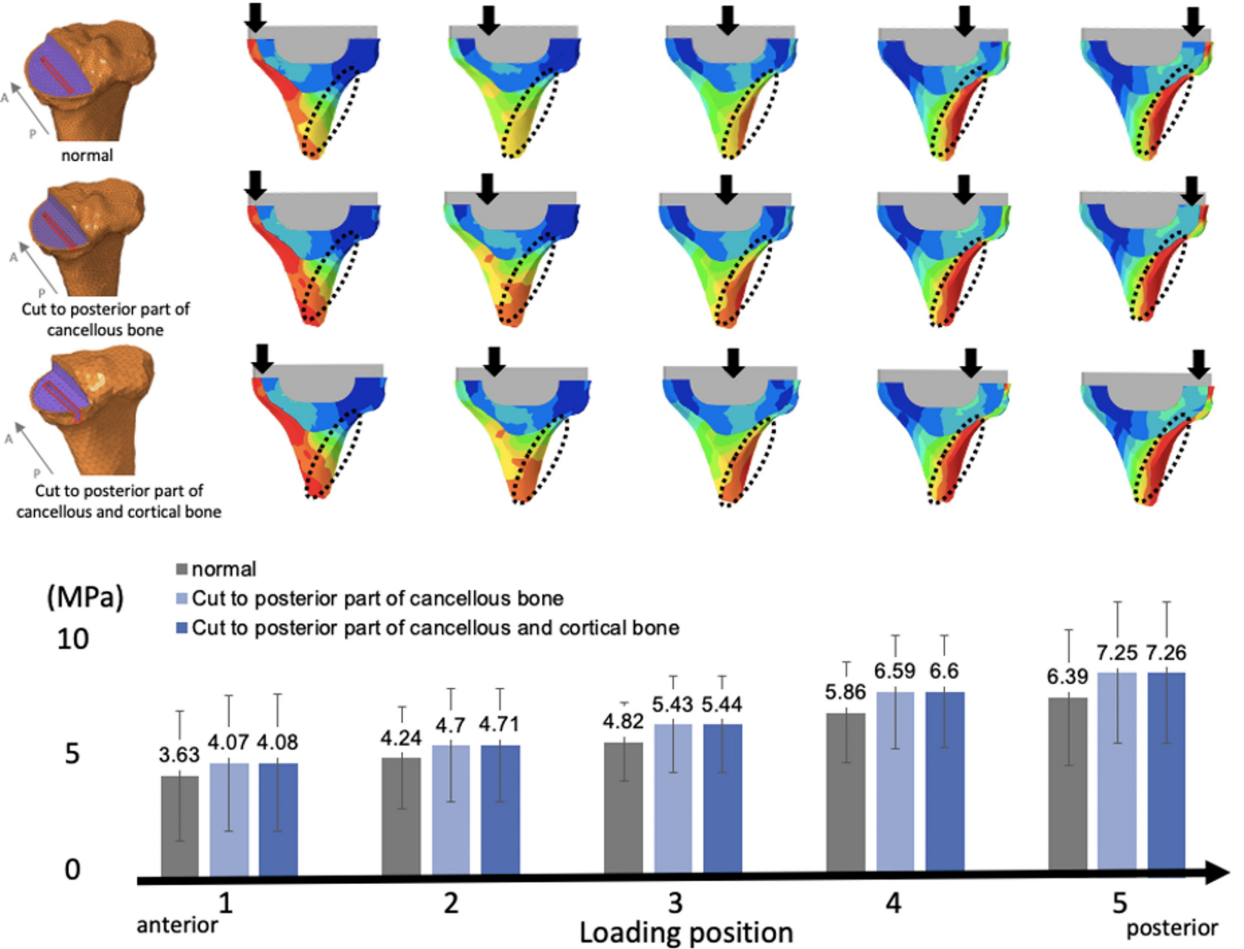
The keel is additionally cut posteriorly (cuts to the posterior part of the cancellous bone case and to the posterior part of the cancellous and cortical bone cases) In both cases, the von Mises stress is increased in the posterior part of the cortical bone of the tibia. The stresses tend to increase when the loading position moves posteriorly.

Finally, when the keel was additionally cut posteriorly, the VMS values in the cortical bone of the posterior tibial diaphysis increased. The stress values were as follows: 4.82 ± 1.47 MPa under normal conditions, 5.43 ± 1.67 MPa when the cut extended to the posterior part of the cancellous bone, and 5.44 ± 1.69 MPa when the cut extended to the posterior parts of both the cancellous and cortical bones. As the loading position moved posteriorly, the stresses tended to increase further (Figure 10).

## Discussion

UKA is less invasive than total knee arthroplasty, preserves the physiological rotation by sparing the ligaments, and is an excellent procedure for early rehabilitation. Consequently, the number of UKA cases has increased worldwide recently [18]. However, with an increase in the number of UKA surgeries, the number of reports on tibial medial condyle fractures has also increased [5]. Most fractures occur in the perioperative period and are associated with intraoperative procedural errors [6].

As reported previously, the causes of medial tibial condyle fractures include tibial implant valgus placement, size mismatch, medial condyle injury due to the osteotomy-guided fixation pin, posterior tibial cortical injury during vertical tibial osteotomy, and tibial implant pressing [6,19,20]. Most of these causes have been attributed to the tibia.

Our findings reveal the distribution of stresses in the sagittal plane of the Oxford UKA system and the influence of various factors on medial tibial condyle fractures. Clinical results showed that the tibial component was more likely to be placed posteriorly and that posterior placement resulted in a shorter distance between the keel and posterior cortical bone. The tibial component is pushed manually anteriorly along the keel during its placement. Therefore, we believe that the tibial component may have been displaced more posteriorly than previously planned. Kamenaga et al. reported that the risk of fracture increased as the distance between the keel and posterior cortical bone decreased. In particular, posterior placement of the tibial component may be a risk factor for fracture [21].

This study also showed that the distance between the keel and posterior cortical bone tended to decrease as the size of the tibial component decreased. Previous studies have reported that bone morphology affects fractures in Asian populations. In particular, the constitutional varus and proximal tibia vara could be risk factors for fractures [22-24]. However, recent literature suggests that Asians are more likely to receive smaller tibial implants and may be at a higher risk of fractures compared to those with standard-sized implants [25,26]. Oxford UKA implants are generally manufactured to fit the Caucasian physique. As Asians generally have relatively smaller tibias than Caucasians, smaller-sized implants are often used. In addition, the anteroposterior length and mediolateral width of the tibial component change with implant size, but the depth and thickness of the keel remain the same [27]. Thus, Asians, who have a bone morphology that fits a small size, tend to have a closer distance between the keel and posterior cortical bone and are at a higher risk of fracture.

**Figure 11:**
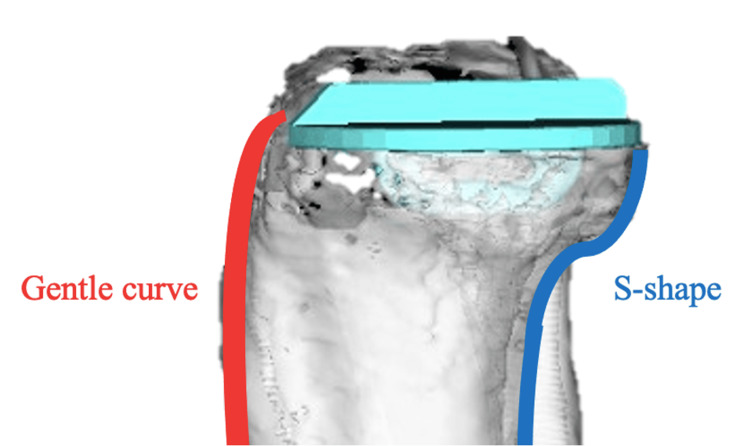
Sagittal image of the proximal tibia The anterior cortical bone is gently curved, while the posterior cortical bone is curved in an S-shape.

The results of the present finite element analysis also showed that the stresses were concentrated in the cancellous bone below the tibial component keel and extended posteriorly to the cortical bone of the tibial diaphysis. We believe that the stress concentration is related to the bony morphology of the tibia. In the sagittal image of the proximal tibia, the anterior cortical bone was parallel to the tibial bone axis, whereas the posterior cortical bone was curved in an S-shape (Figure 11). This distorted bone morphology may be responsible for the stress concentration. In the present study, posterior displacement of the medial loading point and posterior placement of the tibial component increased the stress in the cortical bone of the tibial diaphysis. Inoue et al. noted that the stresses on the posterior cortical bone were considerably higher than those on the medial anterior tibia through finite element analysis of the UKA and reported that this stress concentration caused tibial medial condyle fractures when it exceeded the fracture strength of the cortical bone [4].

In the present study, additional posterior cutting of the keel further increased the posterior tibial stress. For less-experienced surgeons, errors due to posterior sagittal cuts with a narrow operative field are not uncommon [28], and this sagittal bone cut can be the starting point for fractures [29]. The finite element analysis performed in this study showed that various factors, such as posterior displacement of the loading point, posterior placement of the tibial component, and additional posterior cutting of the keel, can cause fractures.

This study utilized a comprehensive analytical approach that combined clinical data and finite element modeling techniques with finite element analysis, which allowed a detailed examination of stress patterns and demonstrated the biomechanical effects of the Oxford UKA keel.

As Oxford UKA is performed with a small skin incision and narrow surgical field, it is difficult to confirm an accurate keel-cut and implant placement. However, based on this study, it is necessary to confirm that excessive keel cuts should not be made and that the tibial components should not be placed posteriorly to prevent tibial medial condylar fractures.

This study had some limitations. First, the finite element analysis is based on a model that simulates real single-patient data. Although efforts have been made to ensure accuracy, individual anatomical differences and specific patient characteristics have not been considered [30]. Furthermore, the analysis focused on the sagittal plane and did not consider the effects of other loading directions or interactions with adjacent anatomical structures. Furthermore, the study primarily investigated the biomechanical aspects and did not directly evaluate the clinical outcomes or long-term implant performance.

## Conclusions

This study sheds light on the stress distribution in the sagittal plane of the Oxford UKA system and the influence of various factors on the tibial bone and implants. This suggests that posterior placement of the tibial components, smaller component size, and an additional cut posterior to the keel may lead to increased stress in the posterior cortical bone, thereby elevating the risk of fractures. The findings of this study will contribute to the understanding of biomechanical considerations in knee arthroplasty and are necessary to optimize surgical techniques and implant designs. In addition, understanding the effects of stresses produced by the Oxford UKA keel from the sagittal plane is essential for the long-term success and stability of knee arthroplasty. Our findings may contribute to optimizing surgical techniques and implant design, ultimately enhancing patient outcomes. These results underscore the critical importance of precise implant placement and surgical techniques to minimize stress concentration and reduce the risk of potential complications. Surgeons should take these findings into account when planning and performing Oxford UKA. Further research is warranted to validate these findings in clinical practice and to explore their impact on long-term implant survival and clinical outcomes.
